# Leakage of an Invagination Pancreaticojejunostomy May Have an Influence on Mortality

**DOI:** 10.1089/pancan.2018.0008

**Published:** 2018-08-01

**Authors:** Harish Lavu, Neal McCall, Scott W. Keith, Elizabeth M. Kilbane, Abhishek D. Parmar, Bruce L. Hall, Henry A. Pitt

**Affiliations:** ^1^ Department of Surgery, Thomas Jefferson University, Philadelphia, Pennsylvania.; ^2^ Department of Biostatistics, Thomas Jefferson University, Philadelphia, Pennsylvania.; ^3^ Indiana University Hospital, IU Health, Surgery, Indianapolis, Indiana.; ^4^ University of Texas Medical Branch, Surgery, Galveston, Texas.; ^5^ Washington University, School of Medicine, St. Louis, Missouri.; ^6^ Lewis Katz School of Medicine at Temple University, Surgery, Philadelphia, Pennsylvania.

**Keywords:** pancreatic adenocarcinoma, pancreatic fistula, pancreaticoduodenectomy, pancreaticojejunostomy

## Abstract

**Purpose:** No consensus exists regarding the most effective form of pancreaticojejunostomy (PJ) following pancreaticoduodenectomy (PD).

**Methods:** Data were gathered through the American College of Surgeons-National Surgical Quality Improvement Program, Pancreatectomy Demonstration Project. A total of 1781 patients underwent a PD at 43 institutions. After appropriate exclusions, 890 patients were analyzed. Patients were divided into duct-to-mucosa (*n* = 734, 82%) and invagination (*n* = 156, 18%) groups and were compared by unadjusted analysis. Type of PJ was included in eight separate morbidity and mortality multivariable analyses.

**Results:** Invagination patients had higher serum albumin (*p* < 0.01) and lower body mass index (*p* < 0.01), were less likely to have a preoperative biliary stent (*p* < 0.01), and were more likely to have a soft gland (*p* < 0.01). PJ anastomosis type was not associated with morbidity but was associated with mortality (duct-to-mucosa vs. invagination, odds ratio = 0.22, *p* < 0.01). Among patients who developed a clinically relevant pancreatic fistula, none of the 119 duct-to-mucosa, compared with 5 of 21 invagination, patients died (*p* < 0.01).

**Conclusion:** Patients who undergo a PJ by duct-to-mucosa or invagination differ with respect to preoperative and intraoperative variables. When an invagination PJ leaks, there may be a greater influence on mortality than when a duct-to-mucosa PJ leaks.

## Introduction

Patients with pancreatic ductal adenocarcinoma (PDA) have an 8% overall 5-year survival rate, with the most effective current therapy being primary tumor resection.^1,2^ Pancreaticoduodenectomy (PD) is the most common surgical procedure performed for resection of PDA; however, this complex operation is associated with a high perioperative complication rate. The most common significant complication associated with PD is a postoperative pancreatic fistula (POPF), which is a leakage of amylase-rich fluid from the site of the pancreaticojejunostomy (PJ).^1,3–5^ Among the three anastomoses performed for pancreatic reconstruction, the PJ is generally considered the “Achilles' heel” of PD, due not only to the relatively high incidence of leakage but also the significance in terms of patient recovery.^6^ Research studies and reviews generally place the incidence of clinically significant POPF to be between 10% and 30%. Although not commonly seen, very severe uncontrolled POPF can even lead to postoperative mortality.^7,8^ Studies have reported mortality rates related to severe POPF ranging from 20% to 40%.^4,9,10^ Given this high risk, an emphasis on decreasing complications associated with the PJ anastomosis is needed to maximize the curative benefit of PD.

Considerable efforts have been made to reduce the incidence of PF in the past 30 years. Fistula mitigation strategies include alternative anastomosis techniques, the use and management of intraperitoneal drains, fibrin glue, and pharmacological agents, among others. None of these methods has proven to be definitively effective.^11–14^ The two standard techniques to restore gastrointestinal continuity for the pancreas are the invagination PJ (IPJ), also known as the “dunking technique,” and the duct-to-mucosa pancreaticojejunostomy (DmPJ). In the IPJ method, the surgeon opens a portion of the jejunum sufficient to “dunk” the pancreatic stump into the side of the jejunum. This technique requires a larger jejunotomy than the competing DmPJ method, where the surgeon makes a small jejunotomy that corresponds only to the size of the pancreatic duct. Sutures are placed directly between the pancreatic duct and the jejunal mucosa, allowing for close adhesion of the two layers.^15–17^ A number of studies have compared the effectiveness of DmPJ and IPJ. One retrospective, single-institution study revealed a 3.2% rate of POPF in the DmPJ group and a rate of 17.5% in its IPJ group without significant mortality differences.^18^ One dual-institution controlled trial, however, found an odds ratio (OR) of 2.4 for POPF incidence between DmPJ and IPJ, again without significant mortality differences.^6^ Still, other studies have reported minimal to no difference in risk of fistula formation or mortality.^15,19,20^ One of the limitations to prior studies is that they were restricted to single or small multi-institutional trials. Given the contradicting conclusions of previous research, more investigation into the effectiveness of these techniques for pancreatic anastomotic reconstruction is warranted.

In this study, we aim to retrospectively compare morbidity and mortality between invagination and duct-to-mucosa PJ from the multi-institutional American College of Surgeons-National Surgical Quality Improvement Program (ACS-NSQIP) Pancreatectomy Demonstration Project (PDP).

## Methods

### Pancreatectomy demonstration project

A multicenter, retrospective cohort study was performed to evaluate morbidity and mortality between invagination and duct-to-mucosa pancreaticojejunostomy. This retrospective analysis was considered exempt by the Institutional Review Board of Thomas Jefferson University. Data were gathered through the ACS-NSQIP, PDP. This initiative gathered pancreatectomy-specific data from 43 participating hospitals (see the Acknowledgments section). Data were collected from November 1, 2011, through December 31, 2012. Data collection was standardized by utilizing trained surgical clinical reviewers (SCRs) within ACS-NSQIP. The SCRs have ongoing data audits and follow precise definitions for data characteristics. Procedures are recorded into the ACS-NSQIP database using Current Procedural Technology codes. These processes have been proven to ensure effective, high-volume data entry into the NSQIP database.^21^ Further details regarding the PDP have previously been published.^22–26^

### Study patients

During the study period, 2805 patients underwent a pancreatic resection at the 43 participating institutions. Patients who underwent total pancreatectomy, distal pancreatectomy, enucleation, and minimally invasive PD were excluded. Patients who lacked data on gland texture and duct size also were excluded, given the significance of these two factors in POPF formation.^27,28^ As a result, 890 patients were available for analysis. Data on patient demographics and comorbidities, preoperative laboratory values, intraoperative characteristics, and postoperative outcomes were collected. Definitions and instructions for data collection of these variables can be found in the ACS-NSQIP Data Users Guide.^29^ Patients were divided into DmPJ and IPJ groups and were compared by unadjusted univariate analyses. Type of PJ was then included in eight separate morbidity and mortality multivariable analyses.

### Outcome measures

All outcomes recorded in the ACS-NSQIP were assessed 30 days postoperatively, except mortality, which could be indicated at any time postdischarge. The primary outcomes of this study were perioperative overall morbidity, serious morbidity as previously defined,^22–26^ mortality, and POPF-associated mortality. Aside from the variables collected by general participation in ACS-NSQIP, the 43 institutions participating in the PDP recorded 24 additional pancreatectomy-specific variables.^22–25^ The PDP-specific variables included in this study were preoperative biliary stenting, chemotherapy and radiation 90 days before PD, pancreatic texture, pancreatic duct size, vascular resection, pancreatic reconstruction method (DmPJ or IPJ), intraperitoneal drain placement, drain removal, drain amylase, delayed gastric emptying, POPF, percutaneous drainage, and presence of malignant histology. ACS-NSQIP PDP definitions have been published in the ACS-NSQIP Procedure Targeted Pancreatectomy Variables and Definitions.^29^ POPF was defined by two scenarios: (1) persistent drain output of amylase-rich fluid of three times the concentration of serum amylase on or after POD 3 in addition to one of the following (drain continuation for longer than 7 days, percutaneous drainage was performed, or reoperation was required) or (2) the clinical diagnosis of POPF by the attending surgeon as well as drain continuation for longer than 7 days, presence of spontaneous wound drainage, percutaneous drainage was performed, or reoperation was required.

### Data analysis

Continuous study variables were summarized between reconstruction groups by their medians and the first and third quartiles and tested for significant difference by Wilcoxon rank sum tests. Categorical study variables were summarized between reconstruction groups by their frequencies and percentages and tested for significant dependencies by Fisher's exact tests. Logistic regression models were used to evaluate the likelihood of binary endpoints (overall mortality during follow-up, overall morbidity, pancreatic fistula, and reoperation) associated with reconstruction (i.e., DmPJ vs. IPJ). Covariates included age (categorized in 10-year groups), body mass index (BMI: kg/m^2^ categorized using World Health Organization guidelines for underweight, normal weight, overweight, obese I, and obese II), sex, preoperative biliary stent, albumin (below vs. ≥3.5 g/dL), texture (soft, intermediate, or hard), and duct size (under 3, 3–6, and >6 mm). Due to the small number of mortality events observed, we used a stepwise selection procedure to identify a model with 3 (or fewer) parameters. The significance level for all tests was 0.05, and all analyses were conducted in SAS version 9.4 (Cary, NC).

## Results

### Study population

During the 14-month study period, 1781 patients underwent PD at 43 participating institutions. After exclusions as outlined above, 890 patients were available for analysis. Of these patients, 735 (82%) underwent DmPJ and 156 (18%) underwent IPJ. Age and gender were similar between DmPJ and IPJ groups (Table 1). The DmPJ patients were found to have a significantly lower median serum albumin (3.8 vs. 4.1 g/dL, *p* < 0.01), higher median serum bilirubin level (0.80 vs. 0.60 g/dL, *p* = 0.02), and higher median BMI (26.2 vs. 24.6, *p* = 0.01). DmPJ patients also were more likely to have a preoperative biliary stent (53% vs. 41%, *p* = 0.01) and were more likely to have undergone neoadjuvant chemotherapy (DmPJ: 9% vs. 4%, *p* = 0.04). Intraoperatively, DmPJ patients had different malignant histologies (84% vs. 79% cancer or intraductal papillary mucinous neoplasm (IPMN), 12% vs. 8% neuroendocrine tumor, *p* < 0.05), but were less likely to have a soft gland (36% vs. 47%, *p* < 0.01; Table 2).

**Table 1.** **Patient Demographics and Preoperative Variables for 890 Patients**

	Duct-to-mucosal	Invagination	*p*
	*n* = 734 (82%)	*n* = 156 (18%)	
Age (years)^a^	65.8 [57.4, 73.1]	65.9 [59.6, 73.4]	0.50
Male	398 (54%)	81 (52%)	0.66
BMI^a^	26.2 [22.9, 30.4]	24.5 [22.3, 28.6]	0.01
Diabetes mellitus	196 (27%)	30 (19%)	0.10
Cigarette smoking	163 (22%)	30 (19%)	0.45
Biliary stent	386 (53%)	64 (41%)	0.01
Preoperative chemotherapy	66 (9%)	6 (4%)	0.04
Preoperative radiation	34 (5%)	2 (1%)	0.07
Albumin (g/dL)^a^	3.8 [3.30, 4.20]	4.1 [3.7, 4.4]	0.01
Bilirubin (g/dL)^a^	0.80 [0.50, 2.0]	0.60 [0.40, 1.80]	0.02
BUN (g/dL)^a^	13 [10, 18]	15 [11, 20]	0.02

^a^ Summarized by median with [first, third quartiles].

BMI, body mass index; BUN, blood urea nitrogen.

**Table 2.** **Intraoperative Variables**

	Duct-to-mucosal, *n* (%)	Invagination, *n* (%)	*p*
Soft gland texture	261 (36)	74 (47)	0.01
Duct size <3 mm	234 (32)	53 (34)	0.78
Vascular resection	90 (13)	19 (13)	1.00
Antecholic DJ/GJ	413 (65)	46 (39)	0.01
Drain placement	577 (81)	137 (92)	0.01
Clean contaminated	609 (83)	148 (95)	0.01
Malignant histology			
CA or IPMN	244 (84)	38 (79)	0.05
Neuroendocrine	34 (12)	4 (8)	
Other	11 (4)	6 (13)	

CA, cancer antigen; DJ/GJ, duodenojejunostomy/gastrojejunostomy; IPMN, intraductal papillary mucinous neoplasm.

### Outcomes

**FIG. 1.**
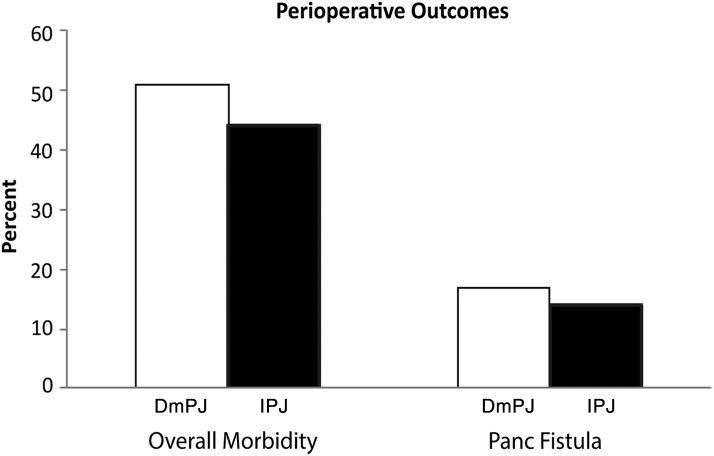
Postoperative outcomes in patients receiving DmPJ vs. IPJ reconstruction. DmPJ, duct-to-mucosa pancreaticojejunostomy; IPJ, invagination pancreaticojejunostomy.

**FIG. 2.**
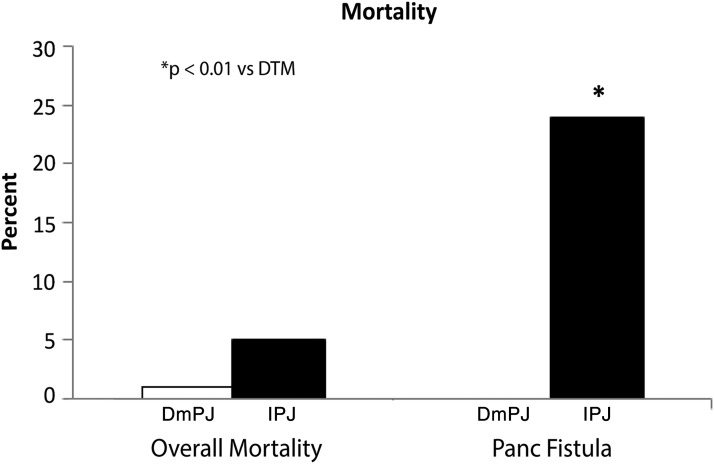
Overall postoperative mortality (among all patients) and mortality following postoperative pancreatic fistula in patients receiving DmPJ vs. IPJ reconstruction.

Overall morbidity and serious morbidity were similar between the two groups (Table 3; Fig. 1). Patients in each group developed complications, including POPF, at similar rates. Both groups also underwent postoperative percutaneous drainage and reoperation at similar rates. However, type of PJ did not influence any of these morbidity outcomes (Tables 3 and 4). On the contrary, overall mortality was higher in invagination patients (1% vs. 5%, *p* = 0.02, Fig. 2). In a logistic regression model adjusted for selected covariates (including sex and albumin indicator as covariates), DmPJ was associated with about a fifth the odds of mortality compared with IPJ (OR = 0.22, 95% confidence interval [0.08–0.64], *p* < 0.01; Table 4). Among patients who developed POPF, none of the 119 DmPJ patients, compared with 5 of 21 invagination patients, suffered postoperative mortality (0% vs. 24% *p* < 0.01; Fig. 2).

**Table 3.** **Perioperative Outcomes**

	Duct-to-mucosal, *n* (%)	Invagination, *n* (%)	*p*
Overall morbidity	372 (51)	68 (44)	0.11
Serious morbidity	323 (44)	58 (37)	0.13
POPF	120 (17)	21 (14)	0.54
Any SSI	160 (22)	29 (19)	0.45
Organ space SSI	67 (9)	18 (12)	0.37
DGE	123 (17)	24 (16)	0.90
DVT	11 (2)	5 (3)	0.18
Percutaneous drain	63 (9)	21 (15)	0.07
Reoperation	19 (3)	6 (4)	0.43
Length of stay^a^	8 [7, 13]	8 [6, 11]	0.13

^a^ Summarized by median with [first, third quartiles].

DGE, delayed gastric emptying; DVT, deep vein thrombosis; POPF, postoperative pancreatic fistula; SSI, surgical-site infection.

**Table 4.** **Logistic Regression Modeling**

	DmPJ vs. IPJ		
Outcome variable	OR	95% CI	*p*
Overall mortality	0.22	(0.08–0.64)	0.01
Overall morbidity	1.22	(0.84–1.76)	0.30
Pancreatic fistula	1.35	(0.78–2.34)	0.28
Reoperation	0.66	(0.26–1.68)	0.38

Each row represents an indicator outcome regressed on a procedure-type indicator (DmPJ = 1, IPJ = 0) and covariates.

CI, confidence interval; DmPJ, duct-to-mucosa pancreaticojejunostomy; IPJ, invagination pancreaticojejunostomy; OR, odds ratio.

## Discussion

POPF is among the most serious complications associated with PD and has broad implications for patient outcomes and recovery from this complex operation. In this multi-institution, retrospective cohort study, we evaluated the influence of the type of PJ on morbidity and mortality. Our analysis suggests an increased risk of overall and POPF-related mortality associated with IPJ compared with DmPJ. These data were adjusted for age, gender, BMI, preoperative albumin levels, and placement of biliary stents, and gland texture and duct diameter via multivariate analysis. The absence of POPF-associated mortality in the DmPJ group compared with the 24% POPF-associated mortality in the IPJ group (*p* < 0.01) suggests that DmPJ may be the safer technique of pancreatic reconstruction.

Surgeon training, judgment, and comfort level are important variables that go into the consideration of the type of PJ anastomosis to perform.^30–32^ Some authorities argue that no single method of PJ can be applied ubiquitously to all patients and that tailoring the method of PJ construction to the patient and the type of gland is the best way to decrease POPF.^10,28^ Small duct diameter and soft pancreatic texture are well documented to increase the risk of complication during pancreatic reconstruction.^6,7,20,30,33,34^

The pathological cause of POPF in instances of small duct diameter and soft pancreatic texture may be fundamentally different depending on the method of reconstruction. Small duct diameter is more likely to lead to improper placement of sutures in the DmPJ anastomosis leading to the potential for distraction of flow across the anastomosis resulting in leakage of pancreatic juice. Whereas in the IPJ anastomosis, the extensive suture placement and necessary compression required to perform the invagination technique in patients with soft pancreatic tissue can cause ischemia, laceration, and dehiscence resulting in a POPF.^28,30,35^ The IPJ also juxtaposes the open end of the pancreatic remnant to the intestinal lumen. This architecture increases the likelihood for enzymatic erosion of the pancreatic tissue, potentially causing necrosis, stenosis, and subsequently POPF.^29,34^ Given the larger opening in the jejunum associated with an IPJ anastomosis, in the event of leakage, bile intermixed with pancreatic juice may lead to further damage of surrounding tissues. This situation may be more likely to result in an organ space infection, sepsis, septic shock, and/or hemorrhage from the gastroduodenal artery stump or other visceral vasculatures.

Studies comparing DmPJ with IPJ after PD have focused primarily on POPF incidence as opposed to fistula-related mortality. In the study by El Nakeeb et al.,^28^ 107 patients were randomized into either end-to-side IPJ or DmPJ groups after pancreatic resection. Patients were followed for 1 year postoperatively, and subsequent analysis revealed no significant difference in POPF between DmPJ and IPJ.^28^ Three mortalities were reported in the DmPJ group and four in the IPJ group, with only some attributable to POPF. No subgroup analysis was carried out on the causes of mortality.^29^

In the largest controlled trial of its type, Berger et al.^6^ randomized 197 patients from two institutions into end-to-side IPJ and DmPJ groups. Analysis revealed a 24% POPF rate in the DmPJ group with a 12% POPF in the IPJ group, and this difference was statistically significant. To our knowledge, this study is the only one to have demonstrated a higher POPF rate with DmPJ. Mortality was low in this study and comparable between reconstruction methods—2% for DmPJ and 0% for IPJ.^6^ Both mortalities in the DmPJ group were directly attributable to POPF. In addition, the study also reported significant differences in POPF rates between institutions as well as individual surgeons.

Based on the lack of a consensus in the literature of the superior reconstruction method, we hypothesized that a larger data set, including a diversity of surgeons and institutions, may reveal additional information regarding POPF incidence, overall mortality, and POPF-associated mortality between DmPJ and IPJ. Previous controlled trials were designed to detect POPF incidence differences between techniques as a primary outcome. Consequently, the largest trial had <100 patients in each group which, given the already low mortality rates at high-volume centers, limited the assessment of mortality between techniques. Our retrospective analysis is the only study to have demonstrated a significantly increased mortality risk in POPF patients undergoing the IPJ technique.

Several limitations are inherent to this retrospective cohort study. First, data were retrieved from many surgeons at many institutions, and studies have reported a wide variation in outcomes based on surgeon skill, experience, volume, and comfort level.^6,26^ However, potential skew in the data was balanced given that the ACS-NSQIP PDP has input from 43 institutions. Second, this nonrandomized retrospective study is subject to selection bias. Whether a particular method of pancreatic reconstruction was selected based on the characteristics of the pancreatic remnant intraoperatively is unknown. For example, surgeons may have reserved one method of reconstruction for patients deemed to be at greater risk of POPF or other complications. The differences in preoperative and intraoperative characteristics may suggest this bias. However, the statistical methodology used in this study controlled for many risk factors, which, in general, would have led to more POPFs in the DmPJ group. The differences between the groups, including the factors of BMI, preoperative biliary stenting, and albumin, would tend to favor the invagination group as it pertains to postoperative complications.

Third, despite multivariate analysis and exclusion of patients without complete data, outcomes measured could have been influenced by confounding factors not recorded in the database. For example, surgeon experience and hospital volume data were not available. Fourth, some parameters measured, such as gland texture, are very difficult to standardize and left to the judgment of the surgeons even with specific ACS-NSQIP definitions. Fifth, with respect to mortality, relatively small numbers are available from which to draw conclusions, and a limitation of the NSQIP database is that we do not have information on the actual cause of death. We can report the association of death with type of anastomosis performed but cannot definitively prove a causal link. The NSQIP database is also limited by the lack of information on pancreatic stents, octreotide, and fibrin glue use. Finally, the DmPJ group was significantly larger (*n* = 734) compared with the IPJ group (*n* = 156), which suggests a national bias and perhaps more experience with this technique.

As for the generalizability of this research, an advantage is that it is the largest study evaluating the two most common methods of pancreatic reconstruction after PD. In the PDP, only 4% of patients had a PG. In addition, a recent multi-institution, prospective randomized trial from Germany suggests that PG is associated with increased postoperative bleeding compared with PJ.^36^ The diversity of the data obtained through the ACS-NSQIP PDP may make this study applicable to hepatopancreatobiliary surgeons both in training and in practice. Given the significant mortality differences between DmPJ and IPJ, future investigation into the safest method for reconstruction should be powered to address difference in mortality as well as POPF.

## Conclusion

This analysis suggests that patients undergoing a PJ by DmPJ or IPJ differ with respect to several preoperative and intraoperative variables. Nevertheless, pancreatic fistula rates did not differ significantly, but mortality was significantly greater with an IPJ, particularly among those experiencing pancreatic fistula. Thus, when an invaginated PJ leaks, there may be a significant impact on mortality.

## Figures and Tables

**Table:** 

Albany Medical Center	Kaiser Permanente SF	University of Alabama
Baptist Memphis	Kaiser Walnut	UC Irvine
Baylor University	Leigh Valley	UC San Diego
Baystate Medical Center	Massachusetts General	University of Iowa
Beth Israel Deaconess	Mayo-Methodist	University of Kentucky
Boston Medical Center	Mayo-St Mary's	University of Minnesota
Brigham and Women's	Northwestern University	University Texas Medical Branch
California Pacific Medical Center	Ohio State University	University of Virginia
Cleveland Clinic	Oregon Health Sciences Center	University of Wisconsin
Emory University	Penn State University	Vanderbilt University
Hospital University Pennsylvania	Providence Portland	Wake Forrest University
Intermountain	Sacred Heart	Wash University St. Louis
IU-University	Stanford University	Winthrop University
IU-Methodist	Tampa General	
Johns Hopkins	Thomas Jefferson University	

## Abbreviations Used

ACS-NSQIP: American College of Surgeons-National Surgical Quality Improvement Program
BMI: body mass index
CI: confidence interval
DGE: delayed gastric emptying
DJ/GJ: duodenojejunostomy/gastrojejunostomy
DmPJ: duct-to-mucosa pancreaticojejunostomy
DVT: deep vein thrombosis
IPJ: invagination pancreaticojejunostomy
OR: odds ratio
PD: pancreaticoduodenectomy
PDA: pancreatic ductal adenocarcinoma
PDP: Pancreatectomy Demonstration Project
PJ: pancreaticojejunostomy
POPF: postoperative pancreatic fistula
SCRs: surgical clinical reviewers
SSI: surgical-site infection
